# Tuberculosis of Patella Complicated by Synovitis of Knee Joint: A Case Report

**DOI:** 10.7759/cureus.24618

**Published:** 2022-04-30

**Authors:** Aadil Khan, Oyovwike S Amedu, Parkash Kumar, Anthony Chukwurah, Olasunkanmi A Kolawole, Ojali R Unedu

**Affiliations:** 1 Department of Surgery, Lala Lajpat Rai Hospital, Kanpur, IND; 2 Family Medicine, Federal Medical Centre Abeokuta, Abeokuta, NGA; 3 Internal Medicine, Liaquat National Hospital and Medical College, Hyderabad, PAK; 4 General Medicine, Apex Specialist Hospital, Awka, NGA; 5 Internal Medicine, University of Texas School of Public Health, Texas City, USA; 6 Internal Medicine, University of Jos, Jos, NGA

**Keywords:** anti-tuberculosis therapy, synovectomy, lymphatics, patella, tuberculosis

## Abstract

Tuberculosis of the patella complicated with synovitis of the knee joint is a rare complication of tuberculosis. Knee joint tuberculosis is usually caused by pulmonary tuberculosis. A few cases are caused by tuberculosis of the digestive tract or lymphatics. Herein, we present a case of a 27-year-old female who presented with left knee pain and swelling that has been managed conservatively with analgesia and hot fomentation over the last two years without improvement. Initial drainage of pus with synovectomy provided improvement. However, anti-tubercular therapy with arthrotomy provided immediate improvement with the resolution of the pain and swelling, and the patient’s gait recovered back to normal. Careful investigation of a patient with prolonged knee pain and swelling is recommended to avoid misdiagnosis with tuberculosis of the patella as a possible differential.

## Introduction

Tuberculosis (TB) is an infectious disease caused by a highly infectious bacterium called *Mycobacterium tuberculosis* [1]. TB can affect any part of the joint, tendon, or bone. The spine is the most affected bone, while tenosynovitis or bursitis occurs in 1% of cases [2]. The occurrence of patellar TB is rare. Due to the rareness of this disease and the variable symptoms, diagnosis is often delayed or challenging as it may present acutely with systemic signs or insidiously with localized symptoms.

Interestingly, the literature reports a 0.09-0.15% incidence of patella TB, despite the knee being the third most affected joint for skeletal TB after the hip and spine [3]. Synovitis is predominantly synovial, with local extension eroding adjacent bone [4]. Imaging is critical in diagnosis because clinical symptoms vary and are often nonspecific. The diagnosis of patellar TB is confirmed by biopsy [5]. When the disease is diagnosed, a combination of surgical treatment and anti-TB drug therapy appears to be curative in treating bone and joint TB [6]. We report a rare case of tuberculous in the patella without other concomitant manifestations. The IRB approval was taken from Ganesh Shankar Vidyarthi Memorial Medical College Ethics Committee (Approval number: EC/BMHR/2022/42).

## Case presentation

A 27-year-old female presented to the outpatient department with complaints of pain and swelling of the left knee joint for the last two years. The pain was acute in onset, progressive and non-radiating; however, the swelling was insidious and progressive. The pain was preceded by intermittent febrile illness, and she also complained about a loss of appetite for the last month. Her past medical history revealed that a local practitioner treated her conservatively with analgesics and hot saline fomentation for left knee pain for the past two years with no improvement. She had no history of trauma and fall. On initial evaluation, she was febrile and vitally stable. On physical examination, the left knee showed ill-defined circumferential swelling, and movement was restricted and painful in the affected limb. Her rest systemic examination findings, including the respiratory system, were unremarkable.

The patient underwent a synovectomy on the posterior surface of the left knee joint to relieve the pain, and the pus was drained to improve knee swelling. She was placed on analgesia. The drained fluid was sent for culture and sensitivity, revealing acid-fast bacilli (AFB) (>10 AFB oil immersions fields), later confirmed as *M. tuberculosis*. She was diagnosed with tubercular arthritis and started on anti-tubercular therapy (Table 1).

**Table 1:** Anti-tubercular therapy for the patient.

Serial number	Dosage form	Drug	Titration on first visit	Titration after correcting dose
1	Tablet	Rifampicin	400 mg once a day	600 mg once a day
2	Tablet	Isoniazid	200 mg once a day	300 mg once a day
3	Tablet	Pyrazinamide	800 mg once a day	1200 mg once a day
4	Tablet	Ethambutol	1200 mg once a day	1500 mg once a day
5	Injection	Streptomycin	750 mg intramuscular	750 mg intramuscular

**Figure 1:**
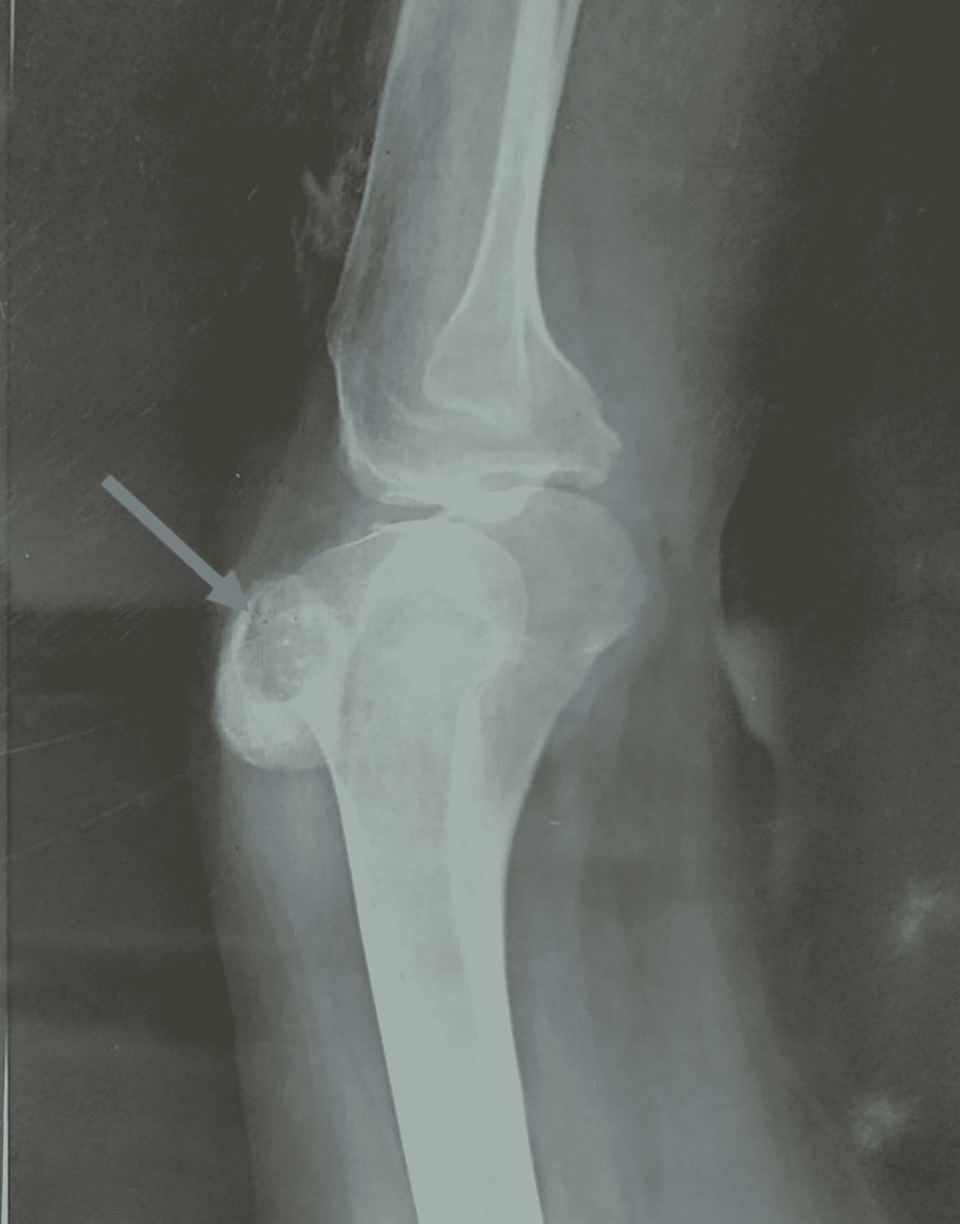
Lateral X-ray of the knee demonstrating localized lytic lesion in the patella with soft-tissue swelling overlying.

**Figure 2:**
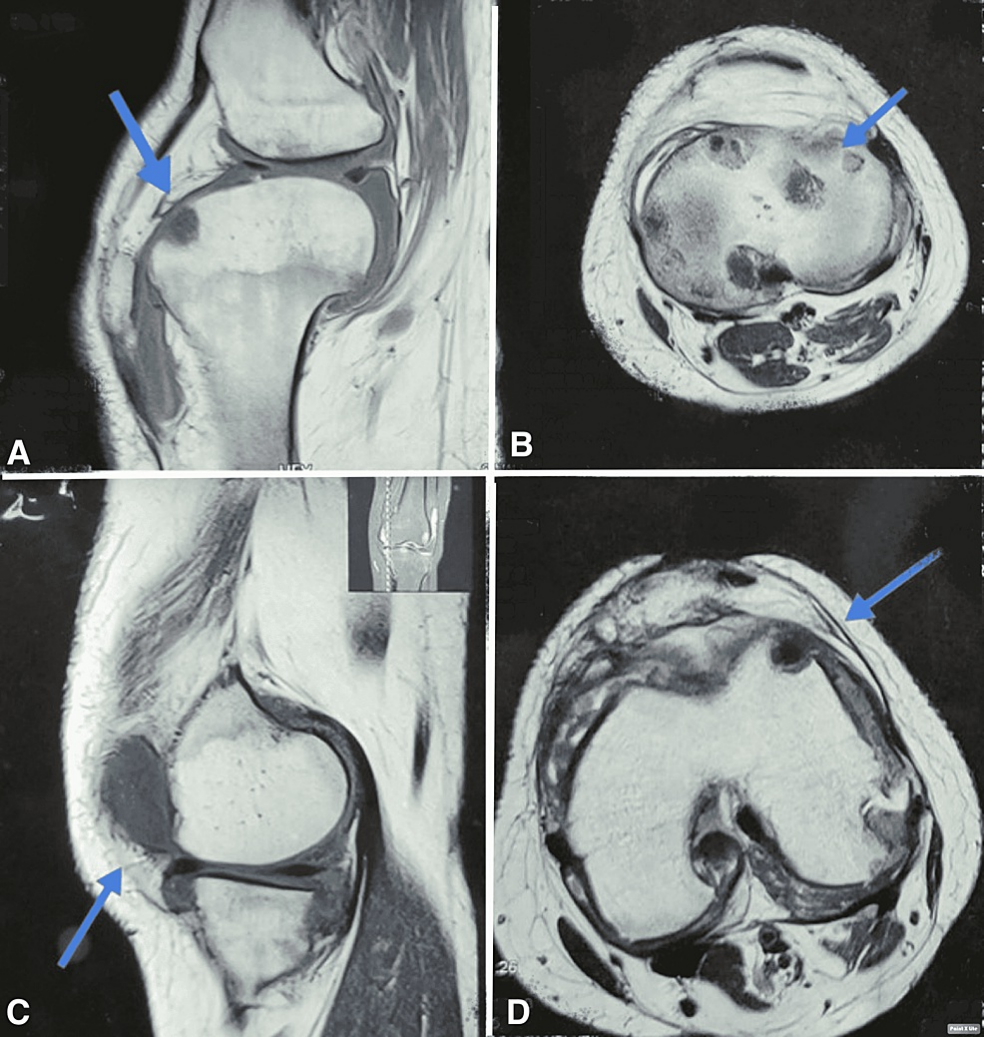
T1/T2-weighted MRI images of knee joint. Coronal sections (A,C) and axial sections (B,D) show well-defined lucency in patella and surrounding area involving cartilage and bony tissue.

However, after two months of follow-up, she came back again with a complaint of severe left knee pain, and her condition worsened. She was admitted to the hospital, and an X-ray knee was performed, which showed cavities in the patella (Figure 1). MRI of the left knee was performed, which showed osteolytic changes in the patella, hyperintensity and edema of the lateral femoral condyle, and remarkable joint effusion at the upper end of the tibia and patellar region was also seen (Figure 2).

Her labs showed increased calcitonin-related peptide (CRP) and erythrocyte sedimentation rate (ESR). Her anti-tuberculosis (ATT) doses were adjusted knowing that she was underdose (Table 1). After that, informed consent for surgery was taken, and a knee arthrotomy for extensive excision and curettage of necrotic tissue from the patella and knee joint was performed, which was done under spinal anesthesia. A left knee tissue biopsy was sent for histopathological examination, which showed extensive ischemic necrosis suggestive of synovial chondrification and cartilaginous degeneration, and underlying stroma showed moderately dense chronic inflammatory infiltrate frequently showing well-formed epithelioid granulomas arranged mostly along synovial lining comprising epithelioid cells, lymphocytes, Langhans type giant cells, and occasional areas of necrosis all impression suggestive of chronic granulomatous tubercular synovitis.

The patient was kept on the cylindrical slab in the affected limb and had proper ATT. Her condition improved and was discharged; however, she was kept under follow-up. The patient showed a marked improved knee function at the four-month follow-up, painless range of motion at the knee joint, and compliance with the anti-tubercular drug without side effects.

## Discussion

Patellar TB is a rare complication of TB. Among skeletal TB sites, the knee is the third most common, with a rate of patella TB of 0.9-0.15% [7,8]. No literature was found that possibly suggests an association between patellar TB and coronavirus infections. The paucibacillary nature of the disease makes it hard to diagnose unless suspicion is very high. Rarity and nonspecific clinical features in the early stages lead to diagnosis delays.

Confirmation of patellar TB is made by tissue biopsy; however, other diagnostic modalities like fluid aspirate for bacteriological evaluation, imaging, and lab parameters can significantly supplement the diagnosis [9,10]. However, past literature suggests that fluid aspirate results are equivocal, and imaging like X-ray and MRI show the local spread of disease, including bursae, synovium, soft tissue, and bone around the knee joint. In our case, remarkable findings were found on T1/T2-weighted MRI images. Serological parameters like ESR and CRP value mostly reveal prognostic value with disease severity and efficacy to treatment.

In our case, the patient developed signs and symptoms of chronic arthritis for the last two years, but the delay in diagnosing tuberculous arthritis led her condition to worsen. The initial symptom of the disease may be nonspecific with absent constitutional symptoms; however, most patients develop chronic knee synovitis. In our patient, synovitis of the knee joint was a remarkable finding.

The treatment varies depending on the severity of the disease. The mainstay treatment for osteoarticular TB is multi-drug ATT for 12-18 months. Surgical cavity curettage and debridement are recommended if there is a persistent, resistant bone lesion, extensive involvement in adjacent areas, or abscess presence [7,11]. In our case, we started the patient first on underdose anti-tubercular therapy, which was increased subsequently. In addition, we performed knee arthrotomy for extensive curettage and debridement of adjacent necrosed areas. The patient responded well to the treatment and was kept under follow-up. At the four-month follow-up, the patient showed a painless full range of motion at the knee joint and was compliant with chemotherapy without any side effects.

## Conclusions

TB of the patella is a rare presentation of TB associated with an unfavorable prognosis, despite the development of treatment options. Due to the high prevalence of TB in developing countries, a preliminary diagnosis of TB can be assumed if imaging, including MRI, reveals unusual marrow signals, synovitis, or joint effusion involving the patella without a history of significant trauma. As a differential diagnosis and management, all surgeons dealing with patients from TB-endemic regions presenting with acute joint swelling should be suspicious of TB.
